# Submillimeter postmortem and in vivo diffusion and susceptibility magnetic resonance imaging to characterize cortical micro- and meso-structures

**DOI:** 10.21203/rs.3.rs-9612742/v1

**Published:** 2026-06-30

**Authors:** Yuto Uchida, Hyeong-Geol Shin, Laura Gomez-Isaza, Juan C. Troncoso, Adnan Bibic, Julianna Gerold, Dongsuk Sung, Yohan Jun, Fuyixue Wang, Zijing Dong, Susie Huang, Berkin Bilgic, Peter van Zijl, Xu Li, Kenichi Oishi

**Affiliations:** 1 Department of Radiology and Radiological Science, Johns Hopkins University School of Medicine, Baltimore, MD 21205, USA; 2 Department of Neurology, Johns Hopkins University School of Medicine, Baltimore, MD 21205, USA.; 3 Department of Biomedical Engineering, Johns Hopkins University, Baltimore, MD 21205, USA.; 4 The F.M. Kirby Research Center for Functional Brain Imaging, Kennedy Krieger Institute, Baltimore, MD 21205, USA; 5 Department of Pathology, Division of Neuropathology, Johns Hopkins University School of Medicine, Baltimore, MD 21205, USA; 6 Athinoula A. Martinos Center for Biomedical Imaging, Massachusetts General Hospital, Boston, MA 02129, USA.; 7 Harvard Medical School, Boston, MA 02115, USA.

**Keywords:** atlas-based analysis, diffusion, fractional anisotropy, human brain, in vivo, postmortem, quantitative susceptibility mapping, magnetic resonance imaging, neuroimaging

## Abstract

The human cerebral cortex consists of anatomically and functionally distinct regions defined by heterogeneous cytoarchitectonic, myelin-related microstructural organization. Quantitative magnetic resonance imaging (MRI) provides a noninvasive framework to resolve subtle regional differences across the cortical mantle. Among different MRI contrasts, diffusion and susceptibility MRI of the brain provide complementary sensitivity to micro-, meso-, and macro-structural organization. However, it remains unclear which fine-scale cortical patterns, typically observed within postmortem scans, are preserved in vivo at matched submillimeter resolution, given limited acquisition time and physiological noise. In this study, we performed a comparative, atlas-based quantification of high-resolution human brain MRI images acquired postmortem and in vivo at approximately 0.5 mm isotropic resolution, focusing on diffusion fractional anisotropy (FA) and tissue magnetic susceptibility (χ) derived from quantitative susceptibility mapping (QSM). For postmortem imaging, diffusion MRI was performed on the left hemisphere from a 71 y/o male using a 3T Connectome 2.0 system, while multi-orientation susceptibility MRI was performed at 7T. In vivo datasets were acquired with diffusion MRI using the signal-to-noise ratio-efficient, distortion-free Romer-EPTI acquisition from a healthy volunteer, 27 y/o female, on the same 3T Connectome, and susceptibility MRI on a 7T system at matched submillimeter resolutions. Using OpenMAP-T1 with the JHU–MNI (Eve) Level 4 parcellation, we performed atlas-based analyses on FA and χ, with additional cortical surface mapping to characterize micro- and meso-structural heterogeneities within the cortical mantle. We then assessed the correspondence between postmortem and in vivo measured metrics. Atlas-based cortical surface mapping revealed consistent anatomical variations in both quantitative MRI metrics, with consistently elevated FA and χ values in the precentral and posterior cortical regions relative to surrounding association cortices, while divergent patterns in the cingulate cortex (higher FA with lower χ values) were also observed. Region-wise comparisons between postmortem and in vivo metrics in the left hemispheric cortical regions demonstrated moderate correspondence for FA and χ values (Pearson’s r = 0.52 [p = 0.02] and 0.53 [p = 0.01], respectively), despite existing biological and technical variations. Together, these findings provide some initial insight into how quantitative diffusion and susceptibility MRI contrast patterns may translate from high-fidelity postmortem imaging to in vivo acquisitions. Combined submillimeter postmortem and in vivo diffusion and susceptibility MRI in the present study can provide a comprehensive anatomical reference for cortical microstructure mapping and support future translations between postmortem and in vivo neuroimaging studies.

## 1. INTRODUCTION

The human cerebral cortex is not a homogeneous sheet of gray matter but is instead composed of distinct regions with characteristic microstructural organization^1^. Since the seminal work of Brodmann, it has been well established that the neocortex can be subdivided into cortical areas based on differences in cytoarchitecture, including variations in laminar structure, neuronal density, and cell morphology^2^. These region-specific microstructural features are closely linked to cortical function, with distinct cytoarchitectonic patterns corresponding to sensory, motor, and associative processing domains^3^. Beyond cytoarchitecture, cortical areas also exhibit systematic differences in intracortical myelination and tissue composition and organization, including myelin content, iron distribution, and microstructural orientation^4^. This heterogeneous microstructural organization forms an anatomical basis for regional functional specialization across the cerebral cortex^5^.

Magnetic resonance imaging (MRI) provides a powerful, noninvasive approach for assessing cortical micro-, meso-, and macro-structures across the entire brain. Recent advances have enabled cortical investigations using diffusion-^6^ and magnetic susceptibility-based MRI contrasts^7^, each offering complementary insights into tissue organization. Fractional anisotropy (FA) from diffusion tensor imaging (DTI) is sensitive to microstructural anisotropy and can reflect the coherence of cortical organization as well as its relationship to underlying white matter pathways^8^. Using this metric, high-resolution diffusion MRI studies have demonstrated depth-dependent and region-specific variations in cortical microstructure, reflecting differences in cytoarchitecture and potentially intracortical myelination across cortical areas. Primary sensorimotor cortices show prominent radial diffusion orientations^9, 10^, while tangential fibers in specific cortical layers, such as layer IV, may also influence the diffusion signal^10, 11^. Tissue magnetic susceptibility (χ) derived from quantitative susceptibility mapping (QSM) provides additional biological information related to magnetic susceptibility sources^12^ and has demonstrated the ability to reveal region-dependent patterns of cortical susceptibility, with age-related increases in sensorimotor as well as frontal, temporal, and parietal association cortices^13^.

However, anatomically comprehensive quantification of small cortical structures remains challenging with current MRI approaches. The thin and highly folded cortical mantle imposes stringent requirements on spatial resolution and signal-to-noise ratio (SNR), necessitating submillimeter imaging that is difficult to achieve uniformly across the entire brain in living subjects^14^. Practical constraints on scan time, sensitivity to motion, and physiological noise further limit the achievable resolution and reliability of in vivo cortical measurements. In comparison, postmortem MRI of human brain tissues overcomes many of these limitations by eliminating subject motion and physiological fluctuations, while allowing prolonged acquisitions under stable imaging conditions and flexible head orientation^15^. These advantages enable substantially higher spatial resolution and SNR than are typically attainable in vivo^16^, providing access to fine-scale neuroanatomical organization and cortical micro- and meso-structures^17, 18^. Accordingly, a wide range of MRI techniques, including DTI^17–20^ and QSM^21, 22^, have been applied to postmortem brain tissues to investigate human brain microstructure and tissue composition. Despite these strengths, the lack of direct, high-resolution correspondence between postmortem and in vivo MRI measurements at the whole-cortex level remains a key gap in translational MRI research. Many postmortem studies have focused on linking MRI contrasts to histological features, yet such investigations are typically confined to selected cortical blocks or limited anatomical regions rather than providing anatomically comprehensive coverage of the cerebral cortex. Consequently, it remains unclear to what extent fine-scale cortical structural features observed in optimized postmortem scans are preserved, attenuated, or spatially redistributed in in vivo MRI, particularly for quantitative contrasts such as FA and χ values.

To address this gap, we conducted atlas-based analyses on FA and χ contrasts using a pair of high-resolution postmortem and in vivo human brain MRI, both at approximately 0.5 mm isotropic resolution. For postmortem MRI, the left hemisphere from a neurologically normal donor was scanned using complementary diffusion and susceptibility MRI protocols at approximately 0.5 mm isotropic resolutions. Postmortem diffusion MRI was acquired with an ex vivo three-dimensional echo-planar imaging (3D-EPI) protocol, and postmortem susceptibility MRI was acquired using multi-orientation gradient-echo (GRE) imaging for calculation of susceptibility through multiple orientation sampling (COSMOS)-based quantitative susceptibility mapping (QSM) reconstruction. For in vivo MRI, diffusion and susceptibility MRI were acquired at matched 0.5 mm isotropic resolutions using Rotating-view EPI-based Time-resolved Imaging (Romer-EPTI)^23^ and GRE at 7T, respectively. This design enabled direct comparison of diffusion and susceptibility contrasts between postmortem and in vivo imaging. We then performed atlas-based analyses assessing whether postmortem regional FA and χ patterns are preserved in vivo despite expected reductions in contrast and sharpness. The objectives of this study were to (i) perform postmortem diffusion MRI and multi-orientation susceptibility MRI of a human brain hemisphere with controlled and reproducible reorientation, both at submillimeter resolutions; (ii) obtain in vivo MRI data with sequences and spatial resolutions matched as closely as possible to the postmortem scans; and (iii) assess the correspondence between postmortem and in vivo cortical microstructural patterns through region-wise, atlas-based comparisons.

## 2. RESULTS

### 2.1 Orientation dependence of GRE tissue frequency map

**Fig. 1:**
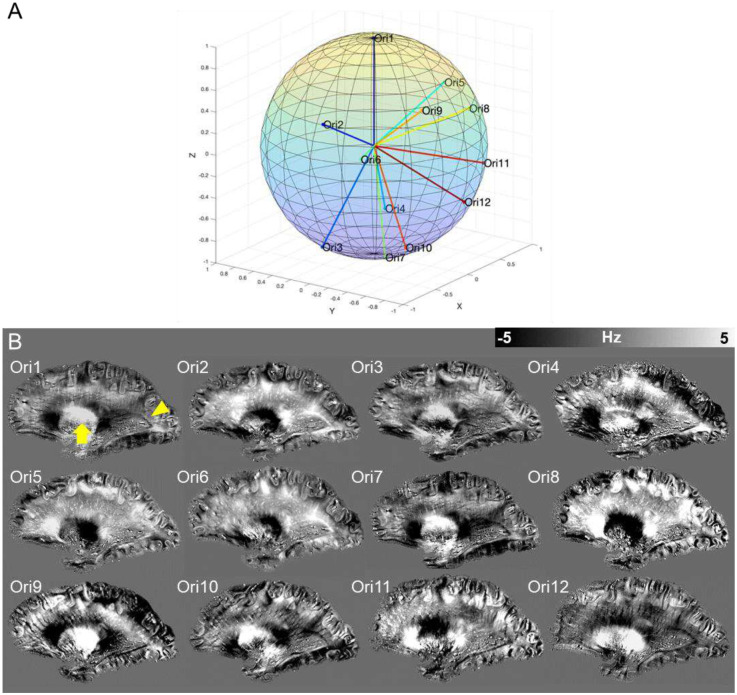
Orientation dependence of postmortem GRE tissue frequency map (A) Gradient echo (GRE) scans acquired at 12 different brain orientations relative to the B_0_ field direction, with unit vectors of the B_0_ field (color-coded lines) in the subject frame of reference spanning uniformly across a half-spherical surface. (B) Tissue frequency maps, showing orientation-dependent contrasts across the brain, e.g., the basal ganglia (yellow arrow) and optic radiation (yellow arrowhead).

Postmortem multi-orientation GRE scans were acquired at 12 different brain orientations relative to the B_0_ field direction. For the co-registered GRE scans, unit vectors of the B_0_ field in the subject frame of reference spanned uniformly across a half-spherical surface (Fig. 1A). Tissue frequency maps demonstrated large orientation dependencies across the brain, especially around the basal ganglia and the major white matter fiber bundles (Fig. 1B), where susceptibility anisotropy effects are also expected to play a role^24–27^.

### 2.2 Atlas-based parcellation of postmortem and in vivo FA and χ maps

**Fig. 2:**
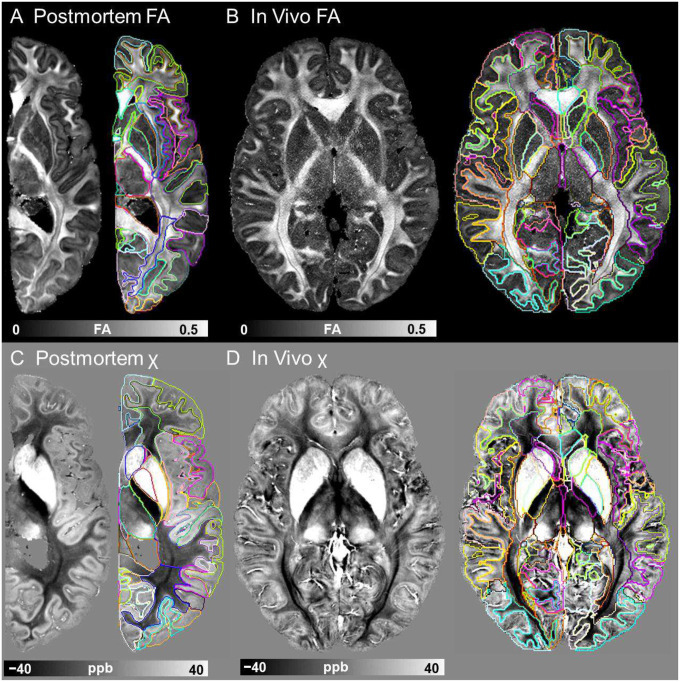
Atlas-based parcellation of postmortem and in vivo FA and χ maps Representative slices show atlas-based parcellation applied to postmortem and in vivo datasets. (A) Postmortem fractional anisotropy (FA) map of the left hemisphere with atlas-based parcellation. (B) In vivo FA map with atlas-based parcellation. (C) Postmortem χ map of the left hemisphere with atlas-based parcellation. (D) In vivo χ map with atlas-based parcellation.

The atlas-based brain parcellation using the JHU–MNI (Eve) atlas (Level 4 parcellation)^28, 29^ through the OpenMAP framework^30^ was applied to both postmortem and in vivo FA and χ data (Fig. 2). The resulting 144 anatomical regions are provided in Supplementary Table 1. In the postmortem data, major cortical and subcortical structures of the left hemisphere were successfully delineated in both datasets, with parcellation boundaries sharply defined, reflecting the high spatial resolution and SNR of the acquisitions. In contrast, in vivo parcellation maps exhibited smoother regional transitions and reduced local contrast, particularly along the cortical mantle and within deep gray matter structures. Despite these differences in spatial sharpness, the same atlas framework could be robustly applied to both FA and χ contrasts, providing a common anatomical basis for subsequent region-wise quantitative comparisons between postmortem and in vivo measurements.

### 2.3 Atlas-based quantification of postmortem and in vivo FA and χ metrics

**Fig. 3:**
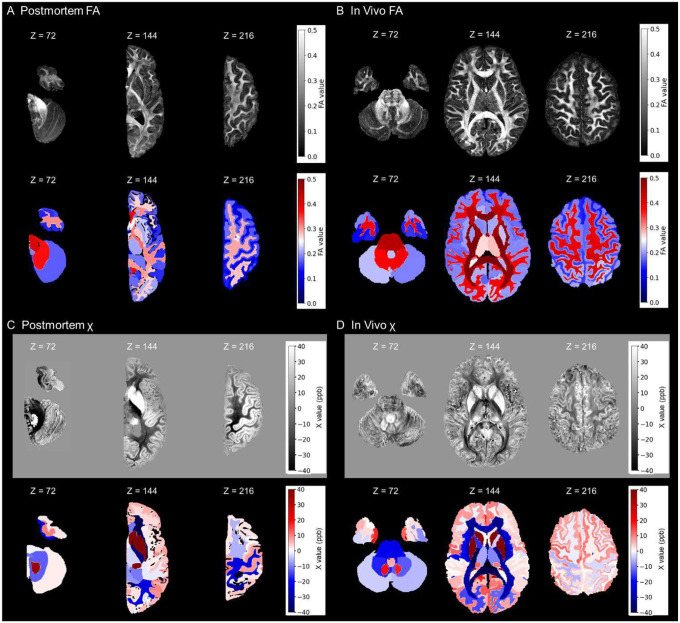
Atlas-based quantification of postmortem and in vivo FA and χ metrics Atlas-based analyses of FA and χ metrics constructed from postmortem and in vivo MRI data. (A) Postmortem FA and its atlas-based analysis of the postmortem human brain hemisphere. (B) In vivo FA and its atlas-based analysis of the healthy volunteer with matched submillimeter resolution. (C) Postmortem χ and its atlas-based analysis of the postmortem human brain hemisphere. (D) In vivo QSM and its atlas-based analysis of the healthy volunteer with matched submillimeter resolution. These quantitative maps are shown in the same anatomical orientation and location for visual comparison.

**Fig. 4:**
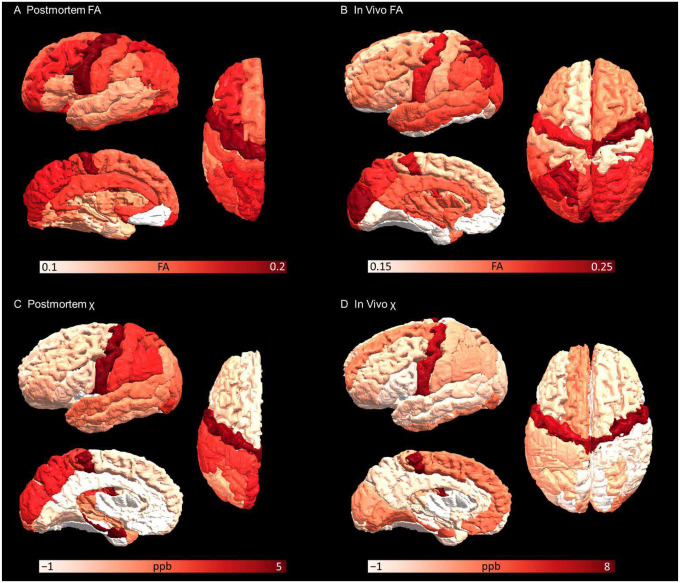
Surface-based visualization of postmortem and in vivo FA and χ metrics Cortical surface maps of FA and χ were generated to highlight regional differences within the cortical mantle. (A) Postmortem FA projected onto the cortical surface. (B) In vivo FA projected onto the cortical surface. (C) Postmortem χ projected onto the cortical surface. (D) In vivo χ projected onto the cortical surface. Surface maps emphasize regional heterogeneity of FA and χ values across cortical areas, with relatively higher FA and χ values in the precentral/postcentral and posterior cortical regions compared with surrounding association cortices. In contrast, the cingulate cortex exhibits relatively high FA but low χ values.

In region-wise analyses of cortical areas, χ metrics demonstrated clear spatial heterogeneity, with relatively higher χ values in the precentral and posterior cortical regions compared with other cortical areas (Fig. 3). FA values were generally low across cortical areas at the whole-brain level, but cortical surface mapping revealed more apparent regional variation within the cortical mantle. Both FA and χ surface maps demonstrated higher values in the precentral/postcentral and posterior cortical regions relative to surrounding association cortices. The cingulate cortex exhibited relatively high FA but low χ values (Fig. 4). Region-wise FA and χ values for all atlas regions, including non-cortical regions such as white matter and deep gray matter nuclei, are provided in Supplementary Table 2. Briefly, FA showed consistently high values across white matter tracts and uniformly low values in deep gray matter nuclei, whereas χ values exhibited limited variation across white matter but marked χ elevations in iron-rich subcortical nuclei.

### 2.4 Quantitative comparisons between postmortem and in vivo FA and χ metrics

**Fig. 5:**
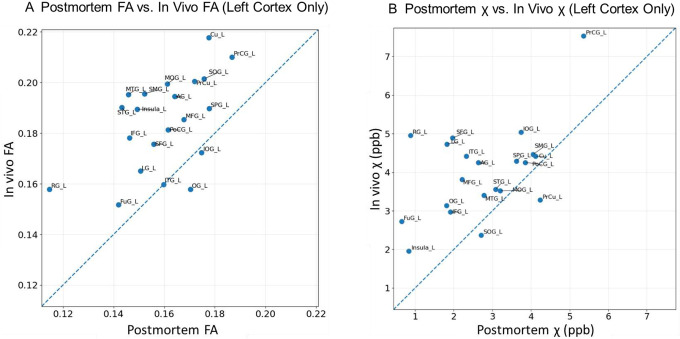
Region-wise comparisons of postmortem vs. in vivo FA and χ metrics in the left hemispheric cortical regions Scatter plots illustrate region-wise comparisons of atlas-derived mean values between postmortem and in vivo datasets using OpenMAP-T1 Level 4 parcellation. FA values demonstrate a significant positive correlation across cortical regions (A: Pearson’s r = 0.52, p = 0.02). χ values show a similar correspondence (B: r = 0.53, p = 0.01). Each data point represents the mean value of a single anatomical region. The dashed line represents the line of identity. For both the FA and χ comparisons, postmortem values were systematically lower than in vivo values across cortical regions, as reflected by the upward shift of most data points above the identity line.

Region-wise FA and χ values were extracted from cortical regions of the left hemisphere for postmortem and in vivo measurements, and their associations were investigated (Fig. 5). Cortical FA values demonstrated a moderate positive correlation between postmortem and in vivo datasets (Pearson’s r = 0.52, p = 0.02), indicating partial preservation of inter-regional anisotropy patterns across the two conditions. Spearman’s rank correlation similarly suggested moderate rank concordance (ρ = 0.49, p = 0.03), supporting the notion that relative regional ordering was partly maintained. Notably, FA values were systematically lower in the postmortem dataset across nearly all cortical regions. For cortical susceptibility, a significant correlation between postmortem and in vivo measurements was also observed (r = 0.53, p = 0.01), whereas Spearman’s rank correlation did not reach statistical significance (ρ = 0.39, p = 0.08), suggesting that relative regional ordering was not consistently preserved between these two datasets. Corresponding results for deep gray matter and white matter areas are provided separately in Supplementary Fig. 1, showing excellent correspondence, especially for χ values of deep gray matter.

### 2.5 Visualization of laminar structures in postmortem and in vivo χ and FA maps

**Fig. 6:**
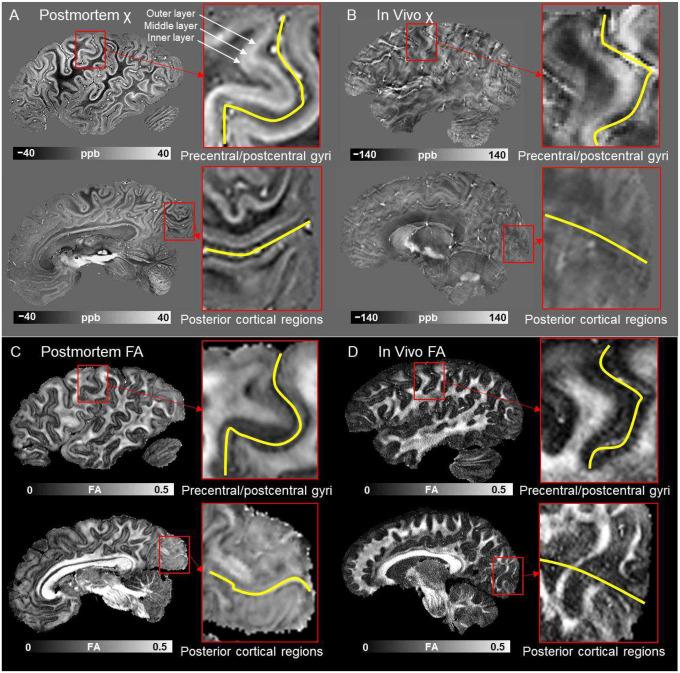
Visualization of precentral/postcentral and posterior cortical regions in postmortem and in vivo χ and FA maps Representative sagittal slices show χ and FA contrasts in the precentral/postcentral and posterior cortical regions. (A) Postmortem χ map demonstrates fine-scale susceptibility variations across cortical layers (outer/middle/inner) in the precentral gyrus, located immediately anterior to the central sulcus (outlined by the yellow contour). In the posterior cortical regions, the calcarine fissure (highlighted by the yellow contour) was examined, where the primary visual cortex extends along both the superior and inferior banks of the fissure. This region provides a well-defined laminar architecture suitable for evaluating susceptibility contrasts. (B) In vivo χ map preserves contrast within the precentral lamination pattern, but visualization in the posterior cortical region is limited due to motion artifacts. (C) Postmortem FA maps demonstrate region-dependent laminar contrast within both precentral/postcentral and posterior cortical areas. (D) In vivo FA maps also reveal cortical lamination, though with reduced sharpness compared with postmortem FA maps.

To illustrate region-specific cortical structural contrasts and to compare laminar visibility between postmortem and in vivo conditions, we focused on the precentral/postcentral cortex, where cortical lamination is relatively well organized, and posterior cortical regions, which exhibit distinct susceptibility and diffusion patterns (Fig. 6). When χ and FA contrasts were examined in the precentral/postcentral and posterior cortical regions, postmortem χ values demonstrated fine-scale susceptibility variations across cortical layers (outer/middle/inner), with clear positive–negative signal transitions. In vivo χ data preserved contrast within the precentral lamination pattern, but visualization in the posterior cortical regions was limited due to motion artifacts. Postmortem FA maps clearly delineated laminar structures within both precentral/postcentral and posterior cortical regions. In vivo FA maps also revealed cortical lamination, despite reduced sharpness compared with postmortem FA maps.

## 3. DISCUSSION

In this study, we performed comparative atlas-based analyses of high-resolution postmortem and in vivo human brain MRI for FA and magnetic susceptibility (*χ*) at approximately 0.5 mm isotropic resolution. Using a custom-built rotatable container, we acquired multi-orientation 7T GRE data from a postmortem human brain hemisphere and reconstructed high-fidelity susceptibility maps. Submillimeter diffusion MRI was also acquired on the same sample using 3T Connectome 2.0. For comparison, in vivo diffusion and susceptibility MRI were acquired from a healthy volunteer at matched submillimeter resolutions but with substantially shorter scan times. In particular, diffusion MRI required approximately 52 hours postmortem versus approximately 3 hours in vivo, likely reducing SNR/CNR and thereby tissue contrast and spatial sharpness in the in vivo data. Despite these limitations, region-wise quantification and cortical surface mapping showed good agreement between postmortem and in vivo measurements for both FA and χ. These findings may help bridge high-resolution postmortem and in vivo MRI, enabling anatomically comprehensive cross-modal comparisons and supporting translational neuroimaging applications.

Susceptibility-based phase contrasts depend on the orientation of tissue microstructures relative to the main magnetic field (B_0_). Multi-orientation QSM methods such as COSMOS combine data acquired at multiple orientations under the assumption of orientation-independent scalar susceptibility to improve the conditioning of the QSM inverse problem^31^. In contrast, susceptibility tensor imaging (STI) explicitly models orientation-dependent susceptibility to estimate susceptibility anisotropy^24–27^. In living subjects, however, head orientation relative to B_0_ can only be adjusted within a limited range, constraining comprehensive orientation sampling. The custom rotatable container enabled controlled and reproducible reorientation of postmortem hemispheres within the 7T system, allowing robust multi-orientation reconstruction at submillimeter resolution. Susceptibility tensor reconstruction is also feasible with the present data, but is beyond the scope of the present comparative study. Our data showed that in vivo QSM preserved key susceptibility contrast patterns observed in postmortem QSM. However, motion, physiological-noise-induced field fluctuation, and limited SNR hindered reliable laminar discrimination in some regions (e.g., posterior cortical regions). This emphasizes the practical challenges of high-resolution, in vivo susceptibility imaging, especially acquired at multi-orientations, and the importance of advanced motion correction strategies, such as navigator-based correction^32, 33^ and dynamic B_0_ field monitoring/correction^34, 35^. These findings also underscore the value of postmortem multi-orientation acquisitions not only as an anatomical ground truth but also as a high-fidelity reference for the training and validation of emerging reconstruction methods—such as physics-informed deep learning approaches—aimed at reducing the required number of orientation sampling for STI^36^.

The distinct cortical patterns observed in the surface-based FA and χ maps may reflect regional variation in the columnar and laminar organization of the human cortex^37^. Cortical minicolumns provide a vertical organizational framework that integrates neuronal somata, dendrites, axonal processes, and local circuits within the cortical mantle, while their structural configuration varies across cortical regions^38^. In the precentral and postcentral cortices, the concurrent elevation of FA and χ values may therefore indicate the coexistence of highly organized intracortical microarchitecture and increased paramagnetic susceptibility sources, respectively. These primary cortices are characterized by pronounced laminar differentiation, dense intracortical myelination, high metabolic demand, and synaptic activity, and have also been associated with relatively high concentrations of non-heme iron. In contrast, the cingulate cortex exhibited relatively high FA but low χ values, indicating a dissociation between diffusion-derived anisotropy and susceptibility-based contrast. Importantly, cortical FA should not be interpreted simply as reflecting myelinated axonal fiber organization; rather, prior work suggests that unmyelinated neurites, including large-caliber apical dendrites, are major contributors to diffusion anisotropy in the cerebral cortex^37, 38^. Therefore, the relatively high FA but low χ in the cingulate cortex may reflect coherent intracortical neurite organization, particularly dendritic and other non-myelinated cellular processes, without a corresponding increase in paramagnetic susceptibility sources. The observed dissociation underscores the importance of combining diffusion- and susceptibility-based contrasts to achieve a more comprehensive characterization of cortical microstructural heterogeneity.

A major goal of this work was to evaluate which diffusion and susceptibility contrast patterns observed postmortem are preserved in vivo at matched submillimeter resolutions. Using the OpenMAP-T1 Level 4 parcellation map, we extracted region-wise FA and *χ* values and found significant correlations between the postmortem and in vivo datasets. While the relative inter-regional ordering of FA and *χ* remained robust across conditions, certain cortical regions (e.g., gyrus rectus) showed measurable deviations between postmortem and in vivo datasets. Notably, postmortem maps showed greater spatial sharpness and tissue contrast, whereas in vivo maps exhibited localized blurring and reduced dynamic range, particularly in *χ* maps near cortical surfaces^39^. These differences are likely at least partly due to imaging artifacts related to motion, physiological noise, and lower SNR due to limited scan time, as well as known factors affecting susceptibility estimation (e.g., fluctuating background fields and imitations in varying brain orientation)^40, 41^. Formalin fixation is also known to alter tissue microstructures and magnetic properties, especially relaxation, through protein cross-linking and dehydration, which may affect diffusivity and, to a lesser extent, susceptibility contrast^42^. In addition, temperature differences between postmortem and in vivo conditions may influence diffusion measurements^43^. Consequently, the lower FA observed postmortem is likely multifactorial, reflecting fixation-induced alterations in tissue microstructure, temperature-dependent diffusivity changes, and differences in SNR that influence tensor estimation.

Differences in age and sex between the postmortem donor and the in vivo volunteer should also be taken into account in the context of known biological influences on iron content, myelin, and tissue microstructures^44^. Aging is associated with region-specific iron accumulation in both subcortical nuclei^45^ and selected cortical regions, including the precuneus and frontal association cortices^46^; therefore, higher *χ* values are anticipated in the older postmortem brain. However, this expected direction was not consistently observed, as several cortical regions exhibited comparable or even lower susceptibility postmortem relative to in vivo. This inconsistency indicates that chronological age alone does not account for the observed patterns and suggests additional contributions from fixation-related alterations in tissue magnetic properties, e.g., slight tissue iron leak, and other potential methodological factors inherent to postmortem susceptibility estimation. Sex differences in iron deposition, with generally higher levels reported in males^47^, also fail to fully explain the regional susceptibility distribution observed here. Similar considerations apply to diffusion metrics, as aging is typically associated with lower FA values^48^, which was observed in the present study. Taken together, part of the divergent regional susceptibility and diffusion MRI patterns observed between postmortem and in vivo datasets may reflect acquisition-dependent factors unique to each imaging condition in addition to biological age- and sex-related differences in iron, myelin, and tissue microstructures.

There are several limitations of the present study. First, the postmortem atlas-based analysis was performed using a single postmortem left hemisphere, and the in vivo analysis was conducted in a single healthy volunteer. Therefore, the findings are likely influenced by inter-individual variability and demographic factors, substantially limiting their generalizability. Expanding the atlas to larger cohorts will be essential to characterize population-level variation and to develop probabilistic reference maps^49^. Second, atlas-based quantification depends on accurate registration and parcellation, which is particularly challenging along the thin and highly folded cortical mantle. Minor misregistration or boundary mismatches may have affected regional mean estimates, especially for small cortical parcels and laminar-sensitive contrasts. Third, the visualization of cortical surface maps was derived from region-wise constant values and global mesh smoothing, which may attenuate fine spatial gradients and laminar-scale variations. Accordingly, surface maps should be interpreted as summaries of regional trends rather than voxel-level representations. Finally, though direct validation against histology was beyond the scope of the present study, integrating histological measures will be a valuable next step to further link diffusion and susceptibility contrasts to underlying microstructural substrates.

In conclusion, we presented a direct atlas-based comparison between high-resolution postmortem and in vivo human brain MR for FA and QSM at approximately 0.5 mm isotropic resolution. Region-specific heterogeneity of cortical FA and QSM contrasts was observed, indicating heterogeneous microstructure organizations across the cortical mantle. Key patterns of FA and QSM metrics postmortem were largely preserved in vivo despite expected reductions in contrast and increased variability. These atlas-based analyses provide a comprehensive reference for studying cortical microstructure, support methodological development in diffusion and susceptibility MRI, and offer a translational bridge between postmortem imaging, in vivo MRI, and future histological validation in neurological and neurodegenerative disease research.

## 4. METHODS

### 4.1 Brain sample and study participant

For the postmortem sample, the left hemibrain of a 71-year-old male was obtained from the Johns Hopkins Brain Resource Center. At autopsy, the fresh brain weighed within 1320 grams, and gross examination revealed no abnormalities. The postmortem study was performed under a protocol for the use of de-identified human brain samples for research purposes, approved by the Institutional Review Board of Johns Hopkins University School of Medicine. For the in vivo experiments, a 27-year-old healthy female volunteer participated in the study. Written informed consent was obtained from the participant prior to participation. The research protocol was reviewed and approved by the Institutional Review Board of Massachusetts General Brigham, and was conducted in accordance with the Declaration of Helsinki.

### 4.2 Postmortem sample preparation and experimental setups

#### 4.2.1 Equipment and materials

The equipment and materials required for setting up the postmortem multi-orientation susceptibility MRI scans are outlined in Supplementary Tables 3 and 4, respectively.

#### 4.2.2 Ellipsoidal brain sample container for multi-orientation susceptibility MRI scans

**Fig. 7:**
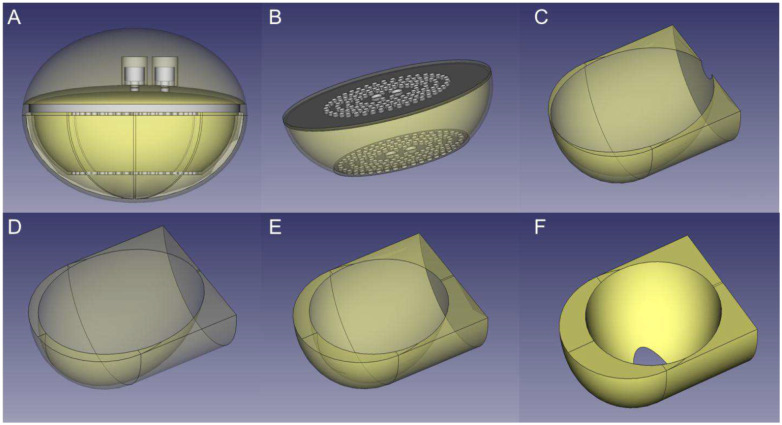
The blueprints of the postmortem hemibrain sample container and the bases used for different brain orientations (A) The whole container. (B) The inner container. (C) The base of 90 degrees. (D) The base of 60 degrees. (E) The base of 30 degrees. (F) The base of 0 degree.

To facilitate the multi-orientation susceptibility MRI scans with controlled rotations along the x (right-left) and y (anterior-posterior) axes, an ellipsoidal container (with two radii of 87 mm and one radius of 112 mm) composed of an outer layer and inner layer to hold each sample tightly, and four bases were designed using FreeCAD (ver. 0.21.0, 2023) and 3D printed (Fig. 7). The bases were designed to fit the 7T head coil and to allow positioning the sample container with its long axis at about 0°, 30°, 60°, 90° with respect to the y axis in the y-z plane (Fig. 7). We printed the models with a layer height of 0.3 mm and a nozzle size of 0.4 mm, ensuring high resolution and accuracy, and used 100% infill to make the container solid and durable. The thickness of the inner layer of the container was 3 mm, and that of the outer layer was 5 mm.

#### 4.2.3 Sample fixation

**Fig. 8:**
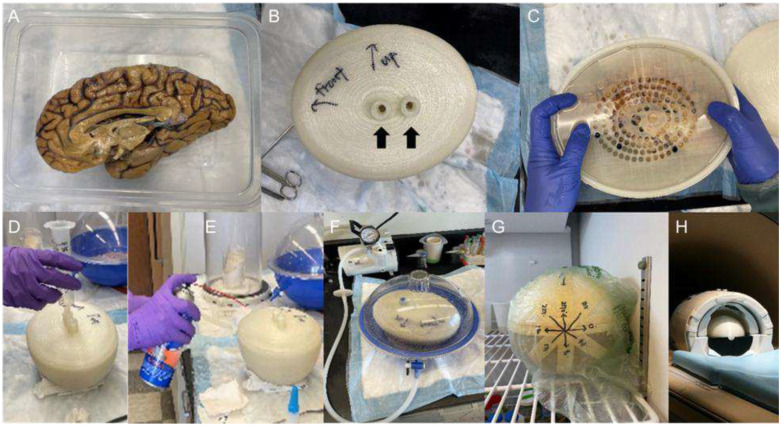
Procedures of brain sample preparation from formalin fixation to MRI scanning (A) The brain sample was transferred to phosphate-buffered saline with 2 mM gadopentetate dimeglumine two days before postmortem MRI scans. (B) The orientation of the brain in the container was marked on the outer lid. Black arrows indicate the top holes through which Fomblin^™^ was poured to the container. (C) The inner lid with plastic pins (black) and pieces was attached to the container to keep the brain sample stable during the MRI scans. (D) Using a 100 ml syringe, Fomblin^™^ was introduced through one of the top holes to fill the container to approximately 80%. (E) The outer lid was pushed to fit the bottom piece with super glue (Gorilla^™^) applied on the contacting surfaces. Spray adhesives (Permatex^™^) were then applied around the container to prevent leakage. (F) The container was placed into a vacuum chamber for more than 12 h, and then Fomblin^™^ was gently added through both holes on the outer lid to fully fill the container. (G) For the multi-orientation acquisition, the brain orientation and the degree of rotations were marked on the container, which was kept in the 35°F refrigerator until the MRI scans. (H) The brain sample was transported to the MRI facility, placed in the head coil, and scanned using the desired MRI pulse sequences.

After the postmortem brain was removed from the skull, the sample was put in 10% formalin solution for 3 months to ensure complete fixation, as performed in previous studies.^50, 51^ Two days before the MRI scans, the brain sample was transferred to phosphate-buffered saline with 2 mM gadopentetate dimeglumine and kept in a 35 F refrigerator (Fig. 8A).

#### 4.2.4 Transferring the brain sample into the ellipsoidal container

The postmortem brain sample was carefully transferred into the ellipsoidal container. The orientation of the brain in the container was marked on the outer lid (Fig. 8B). The inner lid with plastic pins and pieces were attached to the container to keep the brain sample stable during the MRI scans (Fig. 8C). Using a 100 ml syringe, proton-free liquid (Fomblin^™^, Ausimont, Thorofare, NJ, USA) was introduced gently through one of the top holes to fill approximately 80% of the container (Fig. 8D). Remaining formalin or PBS solution floating to the Fomblin^™^ surface was removed using a 1 ml syringe. Then, the outer lid was pushed to fit the bottom piece with super glue (Gorilla^™^) applied on the contacting surfaces between the two pieces. Spray adhesives (Permatex^™^) were then applied around the container to prevent leakage (Fig. 8E). We waited for more than 12 hours until the glue and spray adhesives completely dried.

#### 4.2.5 Vacuuming and sealing procedures

Before transferring the brain sample into the container, large air bubbles trapped in the ventricles were first aspirated using a 27-gauge needle attached to a 1 ml syringe. To further eliminate air bubbles attached to the brain sample and within the container, the sample was placed in a vacuum chamber for more than 12 hours (Fig. 8F). Every three hours during the vacuuming procedure, we gently swayed the container by hand to remove bubbles potentially trapped at certain fissures or gaps around the brain sample.

After the vacuuming process, Fomblin^™^ was gently added through both holes on the outer lid to fully fill the container. Small plastic epoxy plugs were inserted into these holes to prevent unintended leaks or spills in the following steps. A dummy lid was then attached to the container to form the ellipsoid-shaped container, which could be positioned at different angles within the head coil during the MRI scans. To facilitate the multi-orientation acquisition, the brain orientation and the degree of rotations were marked on the container, which was kept in a 35°F refrigerator until any MRI scans (Figs. 8G and 8H). The postmortem sample was moved out of the refrigerator and brought to room temperature at least 6 hours before the MRI scans.

### 4.3 MRI scans

Submillimeter postmortem and in vivo diffusion MRI datasets were acquired on the 3T Connectome 2.0 system^52^ (MAGNETOM Connectom.X, Siemens Healthineers, Forchheim, Germany), using a custom-built 64-channel ex vivo brain coil^53, 54^, with a gradient strength of 500 mT/m and a slew rate of 600 T/m/s.^52, 55^ The postmortem diffusion MRI scan used a segmented 3D EPI sequence (10 segments): TR/TE = 500/40 ms, FOV = 196×138×79 mm^3^, resolution of 0.55 mm isotropic, and 3 b-value shells (b = 3,000, 6,000 and 10,000 s/mm^2^ with 64, 64 and 128 diffusion directions, respectively), δ/Δ = 8/13 ms, TA = 52h 48m. A b0 (b = 0 s/mm^2^) image with reversed phase encoding was also acquired for distortion correction. In vivo diffusion data were acquired using Romer-EPTI^23^ with 0.5 mm isotropic resolution, FOV = 190×192×176 mm^3^, Romer_*factor*_ = 8, MB = 2, 3-shot EPTI^56, 57^, TR/TE = 2080/23 ms, echo spacing = 0.98 ms, 2 b-value shells (b = 1,000 and 2,000 s/mm^2^, with 70 diffusion directions per shell) and 9 b0 images obtained together with each shell, TA = 3h 17m. A 3D T1-weighted image was also acquired on the volunteer using a 0.9 mm isotropic magnetization-prepared rapid gradient-echo (MPRAGE) sequence for anatomical reference.

For susceptibility MRI acquisition, postmortem and in vivo scans were both performed at 7T but using different MRI systems: a Philips 7T MRI system (Philips Healthcare, Best, Netherlands) for postmortem imaging and a MAGNETOM Terra.X 7T MRI system (Siemens Healthineers, Forchheim, Germany) for in vivo imaging. For postmortem, a 3D multi-echo GRE sequence was scanned with the following parameters: TR/TE/ΔTE =47/3/4 ms, 5 unipolar echoes, flip angle = 12°, FOV = 224×180×120 mm^3^, isotropic resolution = 0.5 mm, bandwidth = 503 Hz/voxel, and SENSE acceleration = 2×2, TA=23m 41s. To perform multi-orientation COSMOS-based QSM reconstruction^31^, GRE data were acquired at 12 different orientations of the sample with respect to the B_0_ field (up to 87 of rotation), using the 3D-printed ellipsoidal container as described in Section 4.2.2. In vivo susceptibility MRI was scanned with a similar 3D GRE sequence, 0.5 mm isotropic resolution, TR/TE/ΔTE = 31/3.82/5.43 ms, 5 unipolar echoes, flip angle = 15°, FOV = 256×256×144 mm^3^, bandwidth = 300 Hz/voxel, and GRAPPA acceleration = 3×2, TA=11m 31s. GRE data were acquired at 6 different head orientations relative to the B_0_ field (up to 24° of rotation), enabling COSMOS-based QSM reconstruction.

### 4.4 Data processing

Diffusion MRI data were processed using MRtrix3^58^ to calculate diffusion tensor imaging (DTI) maps. Preprocessing steps include denoising, top-up/eddy correction when applicable (for postmortem data acquired with 3D EPI), and bias field correction. All b-value shells were used for tensor fitting. The resulting FA and T1w MPRAGE images were all co-registered to the corresponding 7T GRE magnitude images (5^th^ echo for postmortem data, 2^nd^ echo for in vivo data), using either rigid transformation for in vivo data or nonlinear warping for postmortem data to compensate for gradient-induced geometric distortion using ANTs^59^. Rigid registration was used for in vivo data to avoid overfitting and preserve native cortical geometry, whereas nonlinear registration was applied to postmortem data to account for different geometric distortions associated with high-resolution diffusion and multi-orientation susceptibility imaging^60^.

Phase images from the 3D ME-GRE data were preprocessed following recent consensus^61^ using best-path-based phase unwrapping, weighted echo averaging for echo combination, and V-SHARP background field removal with a maximum spherical mean value kernel size of 6 mm, to generate the tissue frequency map. The tissue frequency maps at different orientations were rigidly (in vivo) or nonlinearly (postmortem) co-registered to the reference position using ANTs. Multi-orientation QSM data were then reconstructed using the calculation of susceptibility through the multiple orientation sampling (COSMOS) method^31^. The mean susceptibility value of the cerebrospinal fluid in the lateral ventricles was defined as a zero reference^62^.

### 4.5 Atlas-based regional quantification and visualization

For the postmortem dataset, the T1-weighted image of the left hemisphere on 7T was mirrored to generate a synthetic right hemisphere, yielding a bilateral anatomical image. The Open-source Multiple Anatomical Parcellation T1 (OpenMAP-T1; https://github.com/OishiLab/OpenMAP-T1)^30^, a fully automated deep learning-based brain parcellation tool, was then applied to this bilateral T1-weighted image, and the resulting parcellation map was used to extract region-wise quantitative metrics from the postmortem FA and susceptibility maps. For the in vivo dataset, OpenMAP-T1 was applied directly to the 3D T1-weighted image that was coregistered to the same spaces as the FA and susceptibility data. Among the JHU–MNI (Eve) atlases, the Level 4 atlas, comprising 144 regions of interest, was used as the anatomical reference (Supplementary Fig. 2)^28^. The resulting parcellation maps were visually inspected and, when necessary, manually corrected to ensure accurate anatomical correspondence prior to final metrics extraction.

Regional mean values of FA and susceptibility were computed for each anatomical region by averaging voxel-wise values within the atlas-based parcellation maps. For postmortem vs. in vivo comparisons, analyses were restricted to the left hemisphere. Linear associations between regional metrics were assessed using Pearson’s correlation coefficient, while rank-based agreement in regional ordering was evaluated using Spearman’s rank correlation coefficient. To generate atlas-based quantification maps, each area was assigned to its corresponding regional mean value, producing region-wise constant maps for both postmortem and in vivo FA and susceptibility, respectively. These maps were used for visualizing inter-regional contrast patterns across the entire brain. To further delineate differences within the cortical mantle, these region-wise quantitative values were projected onto cortical surface representations using PyVista, a Python-based 3D rendering tool^63^. Cortical surface maps were constructed using a predefined set of cortical regions derived from the OpenMAP-T1 Level 4 parcellation map, including frontal (superior, middle, and inferior frontal gyri), precentral and postcentral gyri, parietal (superior parietal gyrus, supramarginal gyrus, angular gyrus, precuneus), temporal (superior, middle, and inferior temporal gyri, fusiform gyrus), occipital (superior, middle, inferior occipital gyri, cuneus, lingual gyrus), limbic, insula, and cingulate cortices. For each anatomical region, binary masks were generated and converted into surface meshes using the marching cubes algorithm. Individual meshes were assigned spatially uniform scalar values and merged into a single surface representation, followed by global Laplacian smoothing to reduce surface irregularities.

## Supplementary Material

Supplementary Files

This is a list of supplementary files associated with this preprint. Click to download.

- Supplementarymaterial.pdf

## Data Availability

The datasets generated and analyzed during this study are not publicly available due to privacy concerns. However, these data can be obtained from the corresponding author upon reasonable request. Additionally, the custom code used for the analysis in this study is available from the corresponding author upon request.
